# Dr. Michael Talbot

**Published:** 1898-03

**Authors:** 


Dr. Michael Talbot, of Niagara Falls, died at the Buffalo hospital Sisters of Charity Wednesday morning, January 26, 1898, in the 52d year of his age. He had been ill with pneumonia for a few days, but the fatal termination was somewhat sudden and unexpected.

Dr. Talbot was born at Parsonton, King's county, Ireland, May 12, 1846. He received his early education in the schools at Belfast and at the age of 18 he came to America, reaching New York in 1864. Soon afterward he came to Buffalo, spent two years in the school of the Christian Brothers, then entered the Buffalo University Medical College, from which he received his medical degree in 1871. He then was appointed resident physician at the Sisters of Charity hospital and when his term of service expired he located at Suspension Bridge, where he remained until his death. He acquired a large practice and lived to see the village of Suspension Bridge grow until it became part of the city of Niagara Falls.

Dr. Talbot was a member of the New York State Association of Railway Surgeons, of the Medical Society of the County of Niagara and of the Niagara Falls Academy of Medicine. He is survived by a widow and five children : Mrs. Dr. McCarthy, Misses Anna and Stella Talbot and Robert and Frank Talbot, all of Niagara Falls.

